# Two Distinct Populations of α1α6-Containing GABAA-Receptors in Rat Cerebellum

**DOI:** 10.3389/fnsyn.2020.591129

**Published:** 2020-10-06

**Authors:** Petra Scholze, Michael Pökl, Severin Längle, Friederike Steudle, Jure Fabjan, Margot Ernst

**Affiliations:** Department of Pathobiology of the Nervous System, Center for Brain Research, Medical University of Vienna, Vienna, Austria

**Keywords:** cerebellum, GABAA-receptor, receptor composition, radioligand binding, immunoprecipitation

## Abstract

GABAA receptors are pentameric GABA-gated chloride channels. The existence of 19 different subunits (six α, three β, three γ, δ, ε, θ, π, and three ρ) in mammalian systems gives rise to an enormous theoretical diversity of GABAA receptor subtypes with distinct subunit composition and unique pharmacological properties. These receptors are already now the drug targets of several clinically used compounds, such as benzodiazepines, anesthetics, and many more. There is a constant quest to identify novel molecules and possible future drug targets: Currently, α6-containing GABAA receptors are being discussed in the context of treating sensorimotor gating deficits in neuropsychiatric disorders, such as tic disorders and schizophrenia. Therefore, we aim to learn more about α6-containing GABAA receptors. They are mostly expressed in the cerebellar granule cell layer, where they form the following subtypes: α6βxγ2, α1α6βxγ2, α6βxδ, and α1α6βxδ. In former studies, α1α6βxγ2-containing GABAA receptors were considered a single receptor population. In the current study, we investigate the possibility, that this population can consist of two subgroups with alternative arrangements depending if α1 neighbors γ2 (forming a “diazepam-sensitive” receptor), or if α6 neighbors γ2 (forming a “diazepam-insensitive” receptor) and aimed to prove the existence of both subtypes in native tissue. We performed immunoprecipitation experiments on rat cerebellar lysates using α1- or α6 subunit-specific antibodies followed by radioligand binding assays with either ^3^H-flunitrazepam or ^3^H-Ro 15-4513. Indeed, we were able to prove the existence of two distinct populations of α1α6-containing GABAA-receptors and could quantify the different receptor populations: α1βxγ2 receptors constitute approximately 60% of all γ2-containing receptors in the rat cerebellum, α6βxγ2 about 20%, and both isoforms of α1α6βxγ2 9–15% each. The simple classification of GABAA-receptors into αx-containing subtypes seems not to reflect the complexity of nature; those receptors are more diverse than previously thought.

## Introduction

GABAA receptors are ligand-gated chloride ion channels assembled as pentamers of subunits coded by one out of 19 different mammalian genes, grouped into eight classes (α1-α6, β1-β3, γ1–3, δ, ε, θ, π, and ρ1–3; Sieghart, 2015). The existence of receptor pentamers with different subunit composition and arrangement, so-called subtypes, has produced great efforts towards the development of subtype-selective ligands (Sieghart and Savić, 2018). GABAA receptors containing α6-subunits are predominantly expressed in cerebellar granular cells and in the embryologically related granule cells of the cochlear nucleus (Laurie et al., 1992; Wisden et al., 1992; Pirker et al., 2000; Hortnagl et al., 2013). Those α6-containing receptors represent an interesting and diverse population of tonically active extrasynaptic δ- and phasic active synaptic γ2-containing groups. It was suggested that α6 subunits are recruited for the strengthening of synapses and added to α1 subunit containing postsynaptic densities (Accardi et al., 2015). Recent research has identified a number of putative pathophysiological roles which involve α6-containing receptors, and targeting these is considered an interesting strategy for disorders with sensorimotor gating deficits (Chiou et al., 2018), essential tremor (Handforth et al., 2018), and by way of receptors thought to be localized in the trigeminal ganglia, trigeminal neuropathic pain states (Puri et al., 2012; Vasović et al., 2019) and migraine (Fan et al., 2018).

Unlike in the rest of the brain, GABAA receptors in cerebellar granular cells are much less diverse, since only selected subunits are being expressed. In terms of mRNA-abundance, the most significant transcripts are α1, α6, β2, β3, γ2, and δ, with additional weak traces of α4, β1, and γ3 (Laurie et al., 1992; Wisden et al., 1992). These findings are supported by immunohistochemical analysis, indicating protein expression of these subunits in mice (Hortnagl et al., 2013) as well as in rats (Pirker et al., 2000). According to previous results, those subunits assemble to form α1βxγ2, α6βxγ2, α1α6βxγ2, α6βxδ, and α1α6βxδ as major GABAA receptors (Jechlinger et al., 1998; Tretter et al., 2001; Pöltl et al., 2003). The co-existence of the two different α-subunits α1 and α6 in one pentamer has been appreciated previously (Khan et al., 1996), as well as the fact that the benzodiazepine binding pharmacology is defined by the α-subunit, which contacts γ2 and forms the α/γ-interface (Sieghart, 1995). In former studies, α1α6βxγ2-containing GABAA receptors were considered a single receptor population (Jechlinger et al., 1998; Pöltl et al., 2003). However, soon it was appreciated that concatenated α1α6βxγ2 GABAA receptors of two different arrangements can be expressed in *Xenopus laevis* oocytes, either with α1 neighboring γ2 or with α6 neighboring γ2 (Minier and Sigel, 2004). Receptors with such alternative subunit arrangements carry unique drug binding sites. The development of ligands specific for such unique binding sites is facilitated by structural models of the pocket- ligand interactions and has seen a recent surge of experimental models from moderate resolution cryo-EM and crystal structures of heteromeric GABAA receptors (Zhu et al., 2018; Masiulis et al., 2019).

In the current study, we investigated the possibility, that not only in heterologous expression systems but also in the native rat cerebellum, α1α6βxγ2-containing GABAA receptors can consist of two subgroups with alternative arrangements and therefore unique pharmacological properties. Also, we compile current insights gained from ligand-bound α1βγ2 receptors and extrapolate to the α6 containing subtypes described in this study to examine the options for selective targeting of individual receptor species.

## Materials and Methods

### Materials

Rabbit antibodies against GABAA receptor subunits came from a local collection, were all generated as described (Mossier et al., 1994) and characterized in detail and used in several previous studies (Jechlinger et al., 1998; Pöltl et al., 2003; Ogris et al., 2006). Pansorbin^®^ cells were purchased from Merck (Darmstadt, Germany) and Pierce™ BCA protein assay kit from ThermoFisher Scientific (Waltham, MA, USA). ^3^H-flunitrazepam (specific activity 76.0 Ci/mmol) and ^3^H-Ro 15-4513 (specific activity 49.5 Ci/mmol) were purchased from Perkin Elmer NEN (New England Nuclear, Waltham, MA, USA). Diazepam (7-chloro-1,3-dihydro-1-methyl-5-phenyl-2H-1,4,benzodiazepine-2-one) was bought from Nycomed (Opfikon, Switzerland) and Ro15–1788 [Flumazenil, ethyl 8-fluoro-5,6-dihydro-5-methyl-6-oxo-4H-imidazo(1,5-a;1,4)benzodiazepine-3-carboxylate] from Tocris (Bio-techne Ltd., Abingdon, United Kingdom). Standard chemicals were from Sigma-Aldrich (St. Louis, MO, USA).

### Culturing and Transfection of Human Embryonic Kidney 293 (HEK 293) Cells

Human embryonic kidney (HEK) 293 cells (American Type Culture Collection ATCC^®^ CRL-1574TM) were maintained in Dulbecco’s modified Eagle medium (DMEM, high glucose, GlutaMAX™ supplement, Gibco 61965-059, ThermoFisher, Waltham, MA, USA) supplemented with 10% fetal calf serum (Sigma-Aldrich F7524, St. Louis, MO, USA), 100 U/ml Penicillin-Streptomycin (Gibco 15140-122, ThermoFisher, Waltham, MA, USA) and MEM (Non-Essential Amino Acids Gibco 11140-035, ThermoFisher, Waltham, MA, USA) on 10 cm cell culture dishes (Cell+, Sarstedt, Nürnbrecht, Germany) at 37°C and 5% CO_2_.

HEK 293 cells were transfected with cDNAs encoding rat GABAA receptor subunits subcloned into pCI expression vectors. The ratio of plasmids used for transfection with the calcium phosphate precipitation method (Chen and Okayama, 1987) were 3 μg α (1, 2, 3 or 5) : 3 μg β3: 15 μg γ2 per 10 cm dish. The medium was changed 4–6 h after transfection. Cells were harvested 72 h after transfection by scraping into phosphate-buffered saline and pelleted by centrifugation (10 min, 12,000 *g*, 4°C). For membrane binding experiments, cells were resuspended in TC50 (50 mM Tris-Citrate pH = 7.1), homogenized with an ULTRA-TURRAX^®^ (IKA, Staufen, Germany), and centrifuged (20 min, 50,000 *g*). Membranes were washed three times in TC50 as described above and frozen at −20°C until use. For immunoprecipitation assays, cells were lysed in sodium deoxycholate (DOC) buffer (10 mM Tris pH = 8.5, 150 mM NaCl, 0.5% DOC, 0.05% phosphatidylcholine, 0.08% protease inhibitor) at 4°C for 2 h, cleared by centrifugation and used immediately.

### Preparation of Rat Cerebellar Membranes and Extracts

Four to six-week-old female rats were sacrificed by decapitation, the cerebella removed quickly, flash-frozen in liquid nitrogen, and stored at −80°C until needed. For immunoprecipitation experiments, a single cerebellum was thawed at room temperature, homogenized in sodium deoxycholate (DOC) buffer in a brain to buffer ratio of 1 g/10 ml using an Ultra-Turrax rotor-stator homogenizer (IKA, Staufen, Germany) for 10 s followed by syringe and needle homogenization. After incubation (4°C for 2 h) the lysate was cleared by centrifugation and used immediately. For membrane binding experiments rat cerebellum was homogenized with an Ultra-Turrax rotor-stator homogenizer for 30 s in an ice-cold homogenization buffer (10 mM Hepes, 1 mM EDTA, 300 mM Sucrose, protease inhibitor) and centrifuged at 45,000 *g* at 4°C for 30 min. The pellet was resuspended in wash buffer (10 mM Hepes, 1 mM EDTA, protease inhibitor), incubated on ice for 30 min and centrifuged at 45,000 *g* at 4°C for 30 min. The pellet was stored at −80°C o/n and the next day was washed five times by suspension in 50 mM Tris-citrate buffer, pH = 7.1, and subsequent centrifugation as described above. Membrane pellets were stored at −80°C until final use.

### Radioligand Membrane Binding Assays

Frozen membranes were thawed, resuspended, and incubated for 90 min at 4°C in a total of 500 μl of TC50/NaCl (50 mM Tris-Citrate pH = 7.1; 150 mM NaCl), various concentrations of the drug to be studied, 2 nM ^3^H-flunitrazepam or 5 nM ^3^H-Ro 15-4513 in the absence or presence of either 5 μM diazepam or 50 μM Ro 15-1788 (to determine unspecific binding; final DMSO-concentration 0.5%). To study the pharmacology of the α1/γ2-interface membranes were incubated with ^3^H-flunitrazepam; to study the α6/γ2-interface membranes were incubated with 5 nM ^3^H-Ro 15-4513 in the presence of 5 μM diazepam (to saturate α1-containing receptors and target α6-containing receptors only). Membranes were filtered through Whatman GF/B filters and the filters were rinsed twice with 4 ml of ice-cold 50 mM Tris/citrate buffer. Filters were transferred to scintillation vials and subjected to scintillation counting after the addition of 3 ml Rotiszint Eco plus liquid scintillation cocktail.

### Immunoprecipitation Assays

Transfected HEK 293 cells or rat cerebella were lysed in sodium deoxycholate (DOC) buffer (10 mM Tris pH = 8.5, 150 mM NaCl, 0.5% DOC, 0.05% phosphatidylcholine, 0.08% protease inhibitor) at 4°C for 2 h, cleared by centrifugation and used immediately. One hundred and fifty micro litre clear supernatant and 5 μg antibody in 20 μl phosphate-buffered saline (PBS: 10 mM Na_2_HPO_4_, 1.8 mM KH_2_PO_4_, 2.7 mM KCl, 140 mM NaCl, pH = 7.4) were incubated on a shaking platform at 4°C overnight. Heat-killed, formalin-fixed Staphylococcus aureus cells carrying protein A (Standardized Pansorbin-cells, Calbiochem) were centrifuged at 2,300 *g* for 5 min at 4°C. The pellets were washed twice with IP-High (50 mM Tris-HCl pH = 8.3, 600 mM NaCl, 1 mM EDTA, 0.5% Triton X-100), once in IP-Low (50 mM Tris-HCl pH = 8.0, 150 mM NaCl, 1 mM EDTA, 0.2% Triton X-100), and re-suspended with IP-Low. Twenty microlitre of this suspension of Pansorbin cells were added to the above-mentioned cocktail containing the antibody and the solubilized receptors, for 2 h at 4°C on a shaking platform. Samples were centrifuged at 2,300 *g* for 5 min at 4°C and washed twice with IP-High and once with IP-Low at 2,300 *g* for 1 min at 4°C. Pellets were resuspended in 500 μl TC50/NaCl (50 mM Tris-Citrate pH = 7.1; 150 mM NaCl), radioligand was added and the radioligand binding assays performed as described above.

### Data Calculation

Radioligand displacement experiments were analyzed using GraphPad Prism version 8.3.0 for Mac OS X, GraphPad Software^1^, La Jolla, CA, USA. Nonlinear regression analysis of the displacement curves used the equation: Y = Bottom + (Top-Bottom)/(1 + 10^∧^((LogIC50-X)*Hill-slope). IC50 values were converted into Ki values using the Cheng-Prusoff relationship (Cheng and Prusoff, 1973) Ki = IC50/(1 + (S/KD)) with S being the concentration of the radioligand (2 nM for ^3^H-flunitrazepam or 5 nM for ^3^H-Ro 15-4513) and the KD values as described previously [4.8 nM for ^3^H-flunitrazepam (Simeone et al., 2017) and 1.4 nM for ^3^H-Ro 15-4513 in the presence of diazepam (Treven et al., 2018)].

### Structure Rendering and Homology Modeling

PDB files 6HUP and 6D6U were analyzed and rendered with MOE [Molecular Operating Environment (MOE), 2019.01; Chemical Computing Group ULC, 1010 Sherbrooke St. West, Suite #910, Montreal, QC, Canada, H3A 2R7, 2019]. A representative model of an α6+/γ2− the interface is based on 6D6U.

## Results

**Figure 1:**
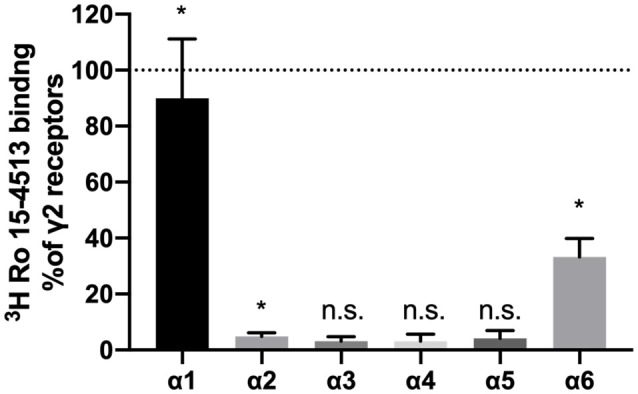
α-subunits found in γ2-containing receptors in rat cerebellum. GABAA receptors were extracted from rat cerebellum, precipitated with subunit specific antibodies, and quantified using ^3^H-Ro 15-4513 binding. Nonspecific binding was analyzed by the addition of 50 μM Ro 15-1788. Results are expressed concerning values obtained with parallel precipitation with specific anti-γ2 antibody. Shown are the mean ± SD of three independent experiments performed in duplicates each. Data were analyzed statistically, if they are significantly different from 0, following a one-sample *t* and Wilcoxon test (*p < 0.05; ns: *p* > 0.05; α1: *p* = 0.0181; α2: *p* = 0.0260; α6: *p* = 0.0129; α3, α4 and α5: *p* > 0.05).

**Figure 2:**
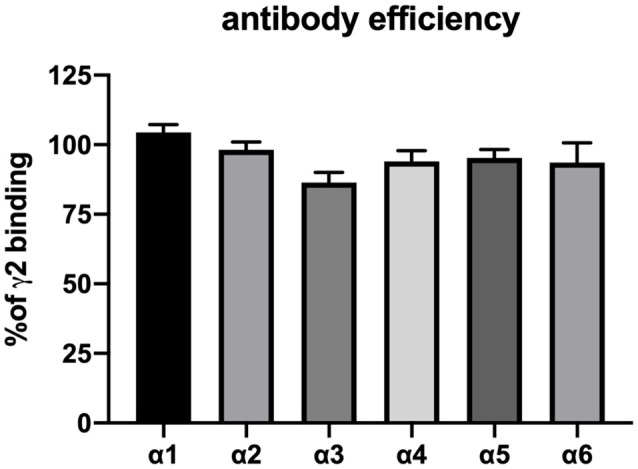
Confirmation of the precipitation efficiency of subunit-specific antibodies used in this study. HEK 293 cells were transfected with the subunit combinations α1βγ2, α2βγ2, α3βγ2, α4βγ2, α5βγ2, and α6βγ2, lysed in deoxycholate (DOC) buffer and precipitated with 5 μg antibody in 20 μl phosphate-buffered saline as described in the “Materials and Methods” section. Precipitated pellets were subjected to radioligand binding assay with 2 nM ^3^H-flunitrazepam (α1βγ2, α2βγ2, α3βγ2, and, α5βγ2) or 5 nM ^3^H-Ro 15-4513 (α4βγ2 and α6βγ2). Nonspecific binding was analyzed by the addition of 5 μM diazepam or 50 μM Ro 15-1788. Results are expressed concerning values obtained with parallel precipitation with specific anti- γ2 antibody. Shown are the mean ± SD of three independent experiments performed in duplicates each. None of the results shown differ statistically from 100% (as determined by a one-sample *t* and Wilcoxon test), indicating that all antibodies precipitate their target receptor with high efficiency.

All ligands used in this study require the presence of the γ2-subunit, and thus, the total pool of γ2-containing GABAA receptors in the cerebellar membrane preparations was subjected to a workflow of differential identification and quantification. The number of γ2-containing GABAA receptors precipitated from rat cerebellar extracts using different α-subunit-specific antibodies was quantified using ^3^H-Ro 15-4513 binding studies and related to the total number of benzodiazepine-sensitive receptors as estimated by precipitation with anti-γ2 antibodies (see Figure 1). Receptors precipitated with α3, α4, and α5 were statistically not significantly different from zero (as determined by a one-sample *t* and Wilcoxon test). Since the precipitation efficiencies of our antibodies have been characterized on transfected HEK 293 cells (see Figure 2), we can conclude that we missed subunits due to poor antibody-quality. Only anti-α1, anti-α6, and anti-α2-antibodies precipitated significant amounts, the latter however only <5% of all receptors. This is in agreement with previous immunocytochemical studies, which found particularly high expression levels of α1 and notable levels of α6 in the cerebellar granule layer, and only weak or absent expression of the other α subunits (Pirker et al., 2000). Those other weakly expressed subunits such as α2 and α5 seem to be predominantly expressed in the molecular or the Purkinje layer of the cerebellum (Pirker et al., 2000; Hortnagl et al., 2013) and the Bergman glia (Wisden et al., 1992), but not in the granule cell layers, where α6 is being expressed. Therefore, when characterizing assembling partners for α6, only α1 will have to be accounted for.

**Figure 3:**
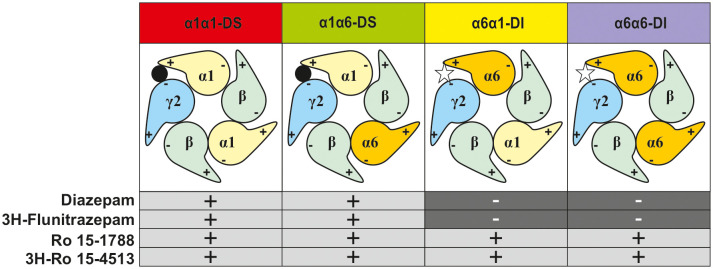
Representation of the possible subtype combinations of γ2-containing GABAA-receptors found in rat cerebellar granule cells and their pharmacological profile. The first named α-subunit is the γ2-neighbor, and thus determines the drug sensitivity profile (diazepam-sensitive “DS” or diazepam-insensitive “DI”).

So indeed, γ2-containing receptors in rat cerebellar granule cells contain α1, α6, and α1α6-subunit combinations. In former studies, α1α6βxγ2-containing GABAA receptors have been considered a single receptor population (Jechlinger et al., 1998; Pöltl et al., 2003). In the current study, we hypothesize that this population consists of two subgroups with alternative arrangements depending if α1 neighbors γ2 (forming a “diazepam-sensitive” receptor, short DS), or if α6 neighbors γ2 (forming a “diazepam-insensitive” receptor, short DI). In the rat cerebellum, we can therefore expect the four different receptor populations depicted in Figure 3.

**Figure 4:**
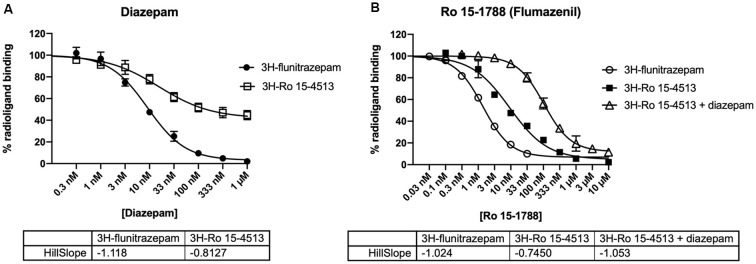
Inhibition of radioligand binding to rat cerebellar membranes. Rat cerebellar membranes were incubated with 2 nM ^3^H-flunitrazepam (circles), 5 nM ^3^H-Ro 15-4513 (squares), or 5 nM ^3^H-Ro 15-4513 + 5 μM diazepam (triangles) in the presence of various concentrations of the displacing ligand [diazepam in panel **(A)** and Ro 15-1788 (flumazenil) in panel **(B)**]. The data shown are the mean ± SD of three independent experiments performed in duplicates each.

To experimentally characterize the different distinct receptor populations found in the native rat cerebellum, radioligand displacement assays were performed. These experiments take advantage of the different subunit selectivity of the two radioligands ^3^H-flunitrazepam, and ^3^H-Ro 15-4513. Although both ligands bind to the benzodiazepine binding site at the α+/γ− -interface (Sieghart, 1995), ^3^H-flunitrazepam, as well as diazepam act *via* the classical “diazepam-sensitive” DS-binding sites at α1βγ2, α2βγ2, α3βγ2, and α5βγ2, while ^3^H-Ro 15-4513 as well as Ro 15-1788 (flumazenil) also bind to the “diazepam-insensitive” DI-binding sites at α4βγ2 and α6βγ2-receptors (Sieghart, 2015), see Figure 3. We performed radioligand displacement assays on rat cerebellar membranes using different radioligands, displacing ligands, and diazepam as DS-specific blocking ligand. First, ^3^H-flunitrazepam was used to selectively label receptors containing an α1/γ-interface (circles in Figures 4A,B). Both compounds diazepam and Ro 15-1788 completely inhibited ^3^H-flunitrazepam binding in a dose-dependent high-affinity one-site binding, as indicated by the Hill-slope of approximately −1. In a second experiment, the radioligand ^3^H-Ro 15-4513 was used to label receptors with any α/γ-interface. Since diazepam only competes for binding at receptors, where α1 neighbors γ2, only partial displacement can be observed (open squares in Figure 4A). Ro 15-1788 on the other hand, can bind to any α/γ-interface, the affinities are however different, depending on if this α is an α1 or an α6. This leads to mixed pharmacology and a dose-response curve with a Hill-slope of −0.7 (closed squares in Figure 4B). To pharmacologically access receptors with an α6/γ-interface, membranes were incubated with ^3^H-Ro 15-4513 in the presence of 5 μM diazepam. Diazepam does not bind to α6βxγ2-receptors under these conditions (data not shown), while Ro 15-1788 concentration-dependently displaced the radioligand in a low-affinity one-site binding with a Hill-slope of −1 (open triangles in Figure 4B).

**Figure 5:**
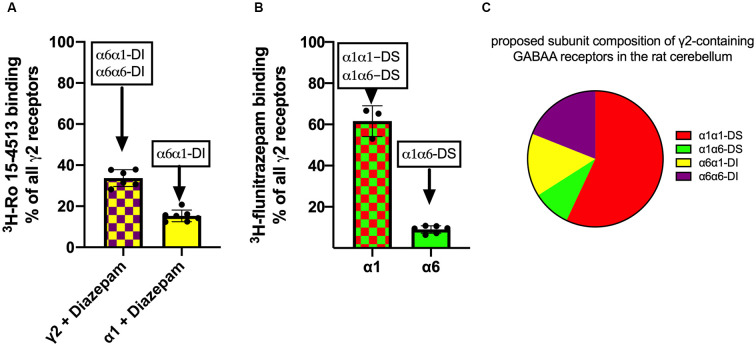
Analysis of γ2-containing receptors in rat cerebellum. GABAA receptors were extracted from rat cerebellum, precipitated with subunit specific antibodies, and quantified using 5 nM ^3^H-Ro 15-4513 with or without diazepam panel **(A)** or 2 nM ^3^H-flunitrazepam panel **(B)**. The data shown are mean ± SD of 4–7 independent experiments performed in duplicates each. Panel **(C)**: the diagram illustrates the proposed subunit composition of γ2-containing GABAA receptors in the rat cerebellum. The pie chart is constructed from the data obtained from experiments depicted in panels **(A,B)**.

Radioligand binding experiments as described in Figure 4 can only give information about the α-subunit which contacts γ2. To gain information on the nature of the other second α-subunit in the pentamer, immunoprecipitation experiments with subtype-specific antibodies were performed, followed by a radioligand binding of the precipitate. Immunoprecipitation of cerebellar lysates with an anti-γ2 antibody followed by an unspecific ^3^H-Ro 15-4513 binding will capture all γ2-containing receptors (“100%”). A similar immunoprecipitation with an anti-γ2 antibody followed by a ^3^H-Ro 15-4513 binding in the presence of 5 μM diazepam will discriminate between diazepam-sensitive receptors with α1 neighboring γ2 (α1α1-DS and α1α6-DS) and the remaining diazepam-insensitive receptors with α6 neighboring γ2 (α6α1-DI and α6α6-DI). The latter proved to be 33.7 ± 1.7% (MW ± SEM, *n* = 6) of all γ2-containing receptors (see Figure 5A). The remaining 66% of receptors will therefore be diazepam-sensitive receptors with α1 neighboring γ2 (the receptors termed α1α1-DS and α1α6-DS). Immunoprecipitation with anti-α1 antibodies followed by an α6/γ2-interface-specific ^3^H-Ro 15-4513 + 5 μM diazepam binding directly measures a receptor population with α6 neighboring γ2 and α1 as second α-subunit in the same pentamer (α6α1-DI and marked in yellow in Figure 3). Those receptors amount to 15.3 ± 1.1% (MW ± SEM, *n* = 7) of all γ2-containing receptors and are depicted as a yellow bar in Figure 5A.

To further analyze the population of diazepam sensitive receptors, with α1 next to γ2, we used immunoprecipitation experiments with subunit specific antibodies, followed by a radioligand binding with ^3^H-flunitrazepam. As determined from the experiments shown in Figure 5A, diazepam-sensitive receptors with α1 neighboring γ2 (α1α1-DS and α1α6-DS) amount to 66% of all γ2-containing receptors. Those receptors will also be measured when immunoprecipitation with an anti-γ2 antibody followed by an α1/γ2-interface specific ^3^H-flunitrazepam binding. The very same receptor population can also be captured when precipitating with anti- α1 antibodies followed by an α1/γ2-interface specific ^3^H-flunitrazepam binding. Immunoprecipitation with anti-α6 antibodies followed by an α1/γ2-interface specific ^3^H-flunitrazepam binding directly measures a receptor population with α1 neighboring γ2 and α6 as second α-subunit in the same pentamer (α1α6-DS, marked in green in Figure 3). Those receptors are depicted as a green bar in Figure 5B and amount to 13.5 ± 1.1% (MW ± SEM, *n* = 6) of all diazepam-sensitive receptors and, since diazepam-sensitive receptors amount to 66% of the total, they account for 9% of total γ2-containing receptors.

With those experiments, we can now define the composition of the bulk of γ2-containing receptors in rat cerebellum (see Figure 5C): 57% α1γ2βxα1βx (=α1α1-DS, red), 9% α1γ2βxα6βx (=α1α6-DS, green), 15% α6γ2βxα1βx (α6α1-DI, yellow), and 19% α6γ2βxα6βx (α6α6-DI, purple).

**Figure 6:**
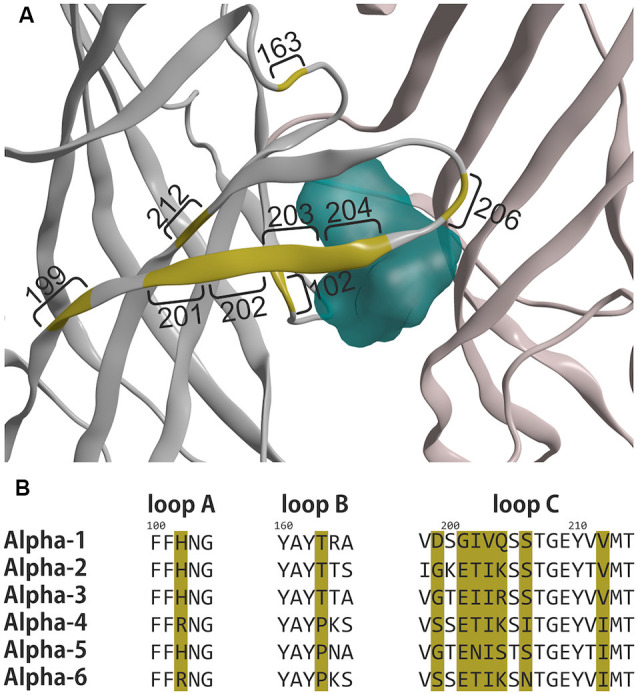
Differences in the principal faces of α1 and α6 subunits. **(A)** Ribbon depiction of the α1+/γ2− ECD binding site from 6HUP. Segments colored in yellow represent amino acids, which differ between α1 and α6. Numbers indicate the number of the amino acid according to the numbering in 6HUP. The molecular surface of diazepam in the pocket is rendered in cyan to indicate the position of the variable amino acids concerning the space occupied by the ligand. **(B)** Partial alignment of sequences containing the different amino acids, depicted in **(A)**. All α isoforms are displayed with the positions of the amino acids, which differ between α1 and α6 colored in yellow. Amino acid numbering corresponds to 6HUP and represents the mature human α1 peptide.

**Figure 7:**
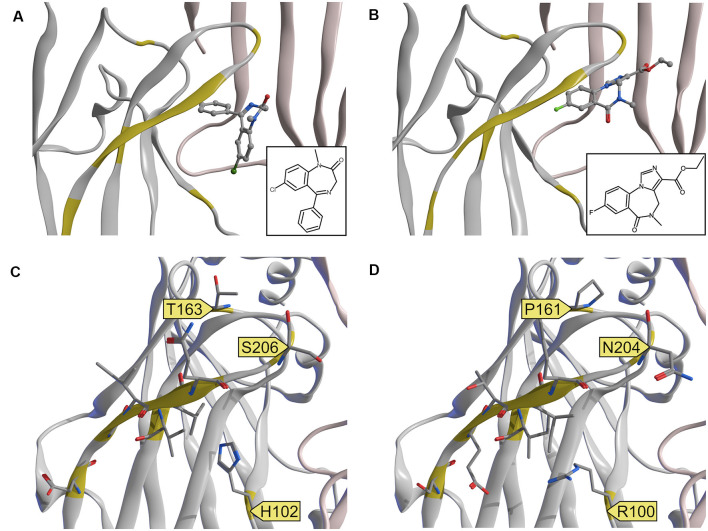
Experimental structures and a homology model of the α6 subunit. **(A,B)** Comparison of experimental structures of diazepam and flumazenil at the α1+/γ2− ECD binding site. 6HUP **(A)** contains diazepam, 6D6U **(B)** contains flumazenil. The two ligands do not share a common binding mode. **(C)** 6HUP and **(D)** α6 homology model, both with amino acids, which differ between the two α isoforms in stick representation. Amino acids that could facilitate DI selective or even α6 selective targeting are highlighted with a yellow flag.

Those receptor subpopulations differ by their pharmacological properties: if ideally selective benzodiazepine-site ligands were available, receptors of the DS-arrangements could be targeted together, as well as those of the DI-arrangements. At this time, substances that interact selectively with DS sites are broadly available and include diazepam and flunitrazepam—and are also used in the current study. In contrast, ligands selective for the α6+/γ2− pocket so far are lacking. Existing experimental structures can provide some guidance towards the design of such ligands, see Figures 6, 7.

A single amino acid on loop A causes the separation between DS and DI subunits, namely His in α1, α2, α3 and α5, and Arg in α4 and α6. A single amino acid in loop B causes zolpidem sensitivity in α1, α2, and α3, and renders α4, α5, and α6 zolpidem insensitive. Loop C displays unique variable amino acids for each α isoform, in theory enabling targeting of each subunit individually. Of all variable amino acids, which differ between α1 and α6, only some contribute directly to the pocket surface, see Figure 7.

## Discussion

Benzodiazepine type compounds have been used clinically as anesthetics, anticonvulsants, anxiolytics, hypnotics, and many more (Sieghart, 2015). They act by allosterically modulating GABAA receptors at the α/γ-interface. Existing experimental structures provide us with information about the molecular binding site of currently used ligands as well as some guidance towards the design of novel, more selective ligands. Computational modeling of potential drug binding sites found on a defined receptor species is however challenging since we lack the required knowledge of the receptor species existing in native tissue. Several receptor isoforms have been described to “exist,” exist with “high probability,” “tentatively” or “most likely” (Olsen and Sieghart, 2008; Mortensen et al., 2012), and this list is constantly being modified. In all of those studies, the nature of the α and γ-subunits is described precisely, there is however a complete lack of knowledge concerning the different β isoforms, which are mostly only listed as βx. Also, it is not fully known, whether all subunits can contribute to all interfaces or if there are assembly rules. It is therefore of uttermost importance to further identify subunit arrangements found in a given native tissue.

In the current study, we mostly focused on receptors containing the α6-subunit, which have gained recent attention as potential novel future drug targets. α6-containing GABAA receptors have been reported to contribute to the alleviation of sensorimotor gating deficits (Chiou et al., 2018) and orofacial pain and migraine (Fan et al., 2018) in animal and cell models. Moreover, models of essential tremor and trigeminal neuropathic pain also have been demonstrated to benefit from drugs that act on α6-containing GABAA receptors (Handforth et al., 2018; Vasović et al., 2019). α6βγ2-containing receptors can be discriminated from α1βγ2, α2βγ2, α3βγ2, and α5βγ2 since they are not sensitive to diazepam. This separation between diazepam-sensitive and diazepam-insensitive subunits is caused by a single amino acid on loop A, namely His in α1, α2, α3 and α5, and Arg in α4 and α6. Interestingly, the experimental structures of diazepam and Ro 15–1788 (flumazenil) in the α1+/γ2− ECD binding site do not indicate a common binding mode of these ligands. Diazepam as a prototypical ligand with selectivity for the DS subunits is close to the loop A residue His102 (see Figure 6A). In the homology model, the unselective flumazenil molecule leaves ample space for the bigger Arg100 sidechain in loop A, based on the unique orientation that was seen in the α1+/γ2− ECD binding site (Zhu et al., 2018). Ligands, which prefer α6 over α1 can theoretically exploit sequence differences not only in loop A, but also take advantage of the loop B difference that renders α6 zolpidem insensitive, and of amino acids unique to the C-loop of α6, see Figures 6B and 7D. Seven amino acids on loop C differ, but two of these do not face the binding site at all. Of the remaining differences, only N204 near the loop tip is unique for the α6 subunit.

The receptors identified in this study could also be discriminated *via* their β-subunits, which may potentially differ in those receptor subtypes. Little is known about the alternative modulatory site which is present at the extracellular α+/β- interface (Ramerstorfer et al., 2011; Varagic et al., 2013a,b; Simeone et al., 2019). We have previously demonstrated that β-isoform-selective ligands can in principle be developed for this site (Simeone et al., 2017). We also have demonstrated that ligands which modulate *via* the α+/β− ECD interfaces can differentiate between α6+/β3− and α1+/β3− (Simeone et al., 2019). To take advantage of these alternative interfaces for selective targeting it would thus be of interest to identify the β-isoforms, which are between the two α subunits in the receptors described here. If they differ among the four receptors we have described here, there is a possibility that selective targeting of individual receptor species is possible. On the other hand, if all these receptors contain the same β isoform in the position between the α subunits, receptors α1α1-DS and α6α1-DI can be targeted together with α6+/β− selective ligands, while receptors α1α6-DS and α6α6-DI can be targeted with α1+/β− selective agents. In order to identify even further possible drug binding sites, we would need experimental structures with more subunits, as well as experimental structures with ligands bound to the α+/β− interfaces.

As mentioned above, for future drug development, it is necessary to precisely identify GABAA receptor subtypes found in a given native tissue. In the current study, we aimed to discriminate the different αβγ2-containing receptors found in rat cerebellum. First, we identified the α-subunits, which exist in the rat brain: α1, α6, and α2, with the latter, only found in <5% of all receptors. This finding is consistent with previous studies: Pöltl et al also find α1 and α6 and also only α2 being significantly different from zero to 7% of total muscimol-binding receptors in the rat (Pöltl et al., 2003). α2-containing receptors, however, seem to be predominantly expressed in the molecular layer (Hortnagl et al., 2013) and the Bergman glia (Wisden et al., 1992), but not in the granule cell layers. Therefore, α2 and α6 although both present in the cerebellum, will not co-assemble. Other studies also describe the (weak) expression of α5 in several different cerebellar cell layers (Pirker et al., 2000). It seems, however, that this subunit does not co-assemble with γ2, which would explain why we were not able to detect significant levels of α5γ2-containing receptors using our experimental conditions. Taking all those findings together, we can therefore expect the following αβγ2-containing receptors in the cerebellar granule cell layer: α1βxγ2, α6βxγ2, α1α6βxγ2. Although most articles only consider αx-containing receptors, the fact that two different α-subunits can co-assemble in one pentamer has been appreciated before: significant, however minor populations of α1α2, α1α3, and α2α3 have been described in the bovine cerebral cortex (Duggan et al., 1991) and α1α6 receptors even in rat brain (Khan et al., 1996). But even those studies do not consider the fact, that the arrangement of the different subunits will crucially influence the inter-subunit binding sites being formed, and therefore heavily influence the pharmacological properties of the receptor isoforms.

In the current study, we, therefore, aimed to further characterize α1α6βxγ2-containing GABAA receptors and hypothesize, that this population consists of two subgroups with alternative arrangements. We identified the γ2-containing GABAA receptors in the rat cerebellum as being composed of 57% α1γ2βxα1βx (=α1α1-DS, red), 9% α1γ2βxα6βx (=α1α6-DS, green), 15% α6γ2βxα1βx (α6α1-DI, yellow), and 19% α6γ2βxα6βx (α6α6-DI, purple; see Figure 5C). These findings are consistent with Ogris et al. (2006), who find that the majority (75%) of all GABAA receptors in the cerebellum contain α1. Among all α6 containing receptors, 30% have been described to contain δ (Jechlinger et al., 1998) but will not be detected using the radioligand binding experiments described in our current study. Jechlinger et al. (1998) describe the remaining non-δ-containing receptors to be distributed to about equal amounts to α6βxγ2 and α1α6βxγ2 (32% related to all α6 containing receptors). This is consistent with our finding, where we find α6α6-DI-receptors (purple segment in Figure 5C) being approximately equal to the sum of α1α6-DS and α6α1-DI (green and yellow segments in Figure 5C). We, however, in addition to these previous studies, can now specify the exact subunit arrangement and can show that indeed both α-positions are occupied by either α1 or α6.

Summarizing, the simple classification of GABAA-receptors into αx-containing subtypes seems not to reflect the complexity of nature; those receptors are more diverse than previously thought.

## Data Availability Statement

All datasets presented in this study are included in the article.

## Ethics Statement

Ethical review and approval was not required for the animal study because The EU directive 210/63/EU, which is also reflected by the Austrian federal law “Tierversuchsgesetz 2012”, states that killing of animals solely for the use of their organs and tissues is not considered a “procedure” and does not require specific approval.

## Author Contributions

PS and ME participated in research design. MP, SL, FS, and JF conducted experiments. MP, SL, and FS performed data analysis. PS and ME wrote or contributed to the writing of the manuscript. All authors contributed to the article and approved the submitted version.

## Conflict of Interest

The authors declare that the research was conducted in the absence of any commercial or financial relationships that could be construed as a potential conflict of interest.

## Footnotes

^1^www.graphpad.com
